# Balanced massive transfusion ratios in multiple injury patients with traumatic brain injury

**DOI:** 10.1186/cc10048

**Published:** 2011-02-22

**Authors:** Sigune Peiniger, Ulrike Nienaber, Rolf Lefering, Maximilian Braun, Arasch Wafaisade, Sebastian Wutzler, Matthew Borgmann, Philip C Spinella, Marc Maegele

**Affiliations:** 1 Department of Trauma and Orthopedic Surgery, University of Witten/Herdecke, Cologne-Merheim Medical Centre (CMMC), Ostmerheimerstrasse 200, D-51109 Cologne, Germany; 2 Institute for Research in Operative Medicine (IFOM), University of Witten/Herdecke, Cologne-Merheim Medical Center (CMMC), Ostmerheimerstrasse 200, D-51109 Cologne, Germany; 3 San Antonio Military Medical Center, 3851 Roger Brooke Drive, San Antonio, TX 78234, USA; 4 Department of Pediatrics, Surgical Critical Care, Department of Surgery, Connecticut Children's Medical Centre (CCMC), 282 Washington Street, Hartford, CT 06106-3322, USA

## Abstract

**Introduction:**

Retrospective studies have demonstrated a potential survival benefit from transfusion strategies using an early and more balanced ratio between fresh frozen plasma (FFP) concentration and packed red blood cell (pRBC) transfusions in patients with acute traumatic coagulopathy requiring massive transfusions. These results have mostly been derived from non-head-injured patients. The aim of the present study was to analyze whether a regime using a high FFP:pRBC transfusion ratio (FFP:pRBC ratio >1:2) would be associated with a similar survival benefit in severely injured patients with traumatic brain injury (TBI) (Abbreviated Injury Scale (AIS) score, head ≥3) as demonstrated for patients without TBI requiring massive transfusion (≥10 U of pRBCs).

**Methods:**

A retrospective analysis of severely injured patients from the Trauma Registry of the *Deutsche Gesellschaft für Unfallchirurgie *(TR-DGU) was conducted. Inclusion criteria were primary admission, age ≥16 years, severe injury (Injury Severity Score (ISS) ≥16) and massive transfusion (≥10 U of pRBCs) from emergency room to intensive care unit (ICU). Patients were subdivided into patients with TBI (AIS score, head ≥3) and patients without TBI (AIS score, head <3), as well as according to the transfusion ratio they had received: high FFP:pRBC ratio (FFP:pRBC ratio >1:2) and low FFP:pRBC ratio (FFP:pRBC ratio ≤1:2). In addition, morbidity and mortality between the two groups were compared.

**Results:**

A total of 1,250 data sets of severely injured patients from the TR-DGU between 2002 and 2008 were analyzed. The mean patient age was 42 years, the majority of patients were male (72.3%), the mean ISS was 41.7 points (±15.4 SD) and the principal mechanism of injury was blunt force trauma (90%). Mortality was statistically lower in the high FFP:pRBC ratio groups versus the low FFP:pRBC ratio groups, regardless of the presence or absence of TBI and across all time points studied (*P *< 0.001). The frequency of sepsis and multiple organ failure did not differ among groups, except for sepsis in patients with TBI who received a high FFP:pRBC ratio transfusion. Other secondary end points such as ventilator-free days, length of stay in the ICU and overall in-hospital length of stay differed significantly between the two study groups, but not when only data for survivors were analyzed.

**Conclusions:**

These results add more detailed knowledge to the concept of a high FFP:pRBC ratio during early aggressive resuscitation, including massive transfusion, to decrease mortality in severely injured patients both with and without accompanying TBI. Future research should be conducted with a larger number of patients to prove these results in a prospective study.

## Introduction

Hemorrhage is one of the main causes of death after trauma. Approximately 40% of severely injured patients die as a result of acute exsanguination [1]. Uncontrolled hemorrhage after trauma is often associated with acidosis, hypothermia and coagulopathy, forming the so-called "lethal triad" [2]. Recently, acute traumatic coagulopathy (ATC) has been identified as a key factor to trigger ongoing hemorrhage after trauma [3]. Ongoing hemorrhage, when combined with ATC, is frequently not controlled by current resuscitation protocols using crystalloids and transfusion of packed red blood cell (pRBC) concentrates [2]. As a consequence, several authors have advocated the early use of fresh frozen plasma (FFP) to control ATC in the early phase after trauma. Despite persistent conflicting data on this issue due to suboptimal methodologies, different retrospective studies have demonstrated a survival benefit of transfusion strategies using an early and more balanced ratio between FFP and pRBC transfusions in patients with ATC requiring massive transfusion within the first 24 hours after hospital admission (≥10 U of pRBCs) [2,4-11]. These analyses, however, have not attempted to determine whether patients with severe traumatic brain injury (TBI) had the same association of improved survival with increased FFP:pRBC ratios. Head-injured patients are particularly prone to developing acute coagulation disorders, and the frequency of these disorders upon emergency room (ER) arrival in patients with blunt TBI has been reported to be as high as 22.7% [12]. In patients with TBI, there is a component of local tissue release of thromboplastin and low platelets. The aim of the present study was to analyze whether a transfusion regimen using a high FFP:pRBC ratio (FFP:pRBC ratio >1:2) would be associated with a similar survival benefit in severely injured patients with TBI (AIS score, head ≥3) as previously demonstrated for patients without TBI requiring massive transfusion (≥10 U of pRBCs).

## Materials and methods

We conducted a retrospective analysis of data from severely injured patients documented in the TraumaRegistry of the *Deutsche Gesellschaft für Unfallchirurgie *(TR-DGU).

### The TraumaRegistry of the *Deutsche Gesellschaft für Unfallchirurgie*

The TR-DGU was founded in 1993 by the German Society of Trauma Surgery (Deutsche Gesellschaft für Unfallchirurgie (DGU)). It is a prospective, multicenter, standardized and anonymous documentation of multiply injured trauma patients at four consecutive posttrauma phases from injury to hospital discharge: (1) prehospital phase, (2) emergency room and initial surgery (until admission to the intensive care unit (ICU)), (3) ICU and (4) outcome status at discharge and description of injuries and procedures. The registry contains detailed information on patient demographics; injury patterns; comorbidities; pre- and in-hospital management; time course; relevant laboratory findings, including data on transfusions; and the outcome of each patient. All injuries are coded using the Abbreviated Injury Scale (AIS). Through 2008 data from a total of 42,248 trauma patients had been entered into the registry, with approximately 6,000 new cases added each year. Since the introduction of the online version of the registry in 2002, the use of FFP has routinely been documented. Between 2002 and 2008, 31,124 patients were entered into the registry. There are 166 hospitals affiliated with the registry, mostly in Germany (*n *= 107), of which 116 actually contribute data to the database. Contributing hospitals are mostly level I trauma centers. The data are not dominated by single trauma centers, but this does not exclude potential center effects due to different levels and strategies of trauma care. The TR-DGU is a voluntary registry, and participation is free of charge. The trauma registry is approved by the review board of the German Society of Trauma Surgery (DGU) and is in compliance with the institutional requirements. As the TR-DGU is an anonymous registry, the Institutional Review Board waived the need for informed consent.

### Data analysis

**Figure 1:**
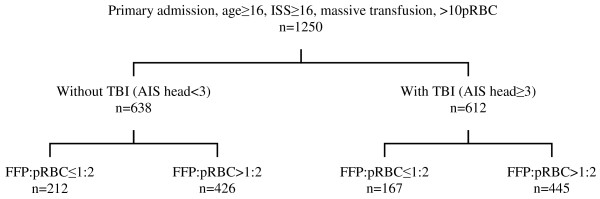
**Flowchart of analyzed patients**. pRBC, packed red blood cell; ISS, Injury Severity Score; TBI, traumatic brain injury; AIS, Abbreviated Injury Scale; FFP, fresh frozen plasma.

Inclusion criteria for the present analysis were primary admission, patient age ≥16 years, severe injury (ISS ≥16) and massive transfusion (≥10 U of pRBCs) from the ER until ICU admission. Patients who died within the first hour after admission were excluded. Patients were subdivided into patients with TBI (AIS score, head ≥3) and patients without TBI (AIS score, head <3) and according to the ratio of plasma to blood that they received during the initial massive transfusion: high (FFP:pRBC ratio >1:2) or low (FFP:pRBC ratio ≤1:2). The FFP:pRBC ratio distribution is shown in Figure 1. All transfused FFP was fresh and frozen (that is, no thawed plasma). Only pRBCs and FFP that had been transfused between ER and ICU admissions were considered. The mean time (± standard deviation (SD)) from ER arrival to ICU admission, including emergency operative procedure time, was 331 ± 144 minutes. The mean time (±SD) required for initial diagnostic procedures and treatment in the ER was 65 ± 42 minutes.

The primary outcome was mortality (6 hours, 24 hours 30 days as well as overall in-hospital mortality). Secondary outcomes included sepsis (defined by Bone *et al*. [13] as adequate symptoms in combination with evidence of germs), multiorgan failure (defined by Sepsis-Related Organ Failure Assessment score ≥3), ventilator-free days (calculated for a total of 30 days), ICU length of stay and hospital length of stay (HLOS).

### Statistical analysis

Demographic and clinical data are presented as means ± SD for continuous variables and as percentages for categorical variables. For continuous variables, normal distributions were analyzed by using the Shapiro-Wilk test. To detect differences between the patient groups, Student's *t*-test or the Mann-Whitney *U *test was performed, depending on the underlying distribution. For categorical variables, a χ^2 ^test was used. Thirty-day survival rates are presented using Kaplan-Meier curves, and differences among the subgroups with and without TBI were tested using the log-rank test.

Multivariate logistic regression analysis was performed with hospital mortality as the dependent variable. Results are presented as odds ratios (ORs) with 95% confidence intervals (95% CIs). The following variables were considered as independent predictors: (1) transfusion ratio (FFP:pRBC ratio ≤1:2 and FFP:pRBC ratio >1:2), (2) Revised Injury Severity Classification (RISC) score as a summary measure for known risk factors for mortality and (3) emergency operations. The RISC score was developed on the basis of data from the TR-DGU to calculate the probability of death for individual patients on the basis of the following 11 variables: age, new ISS, head injury, severe injuries of the extremities, Glasgow Coma Scale score, partial thromboplastin time, base excess, cardiac arrest and indirect signs of bleeding (systolic blood pressure <90 mmHg, hemoglobin <9 g/dl and massive transfusion during initial resuscitation) [14]. A comparison with other established scores (for example, Revised Trauma Score and Trauma Injury Severity Score) demonstrated the superior precision, discrimination and calibration of the RISC score. Statistics were calculated using the SPSS version 18 software package (SPSS, Inc., Chicago, IL, USA).

## Results

A total of 1,250 data sets of severely injured patients derived from the TR-DGU between 2002 and 2008 were eligible for analysis. The mean patient age (±SD) was 41.8 ± 16.3 years, the vast majority of patients were male (72.3%), the mean injury severity (±SD) reflected by ISS was 41.7 ± 15.4 points and the mechanism of injury was predominantly blunt force trauma (90%). Patients were divided into four subgroups as described above and as shown in Figure 1. Table 1 summarizes the demographic and physiological characteristics of patients within their respective subgroups. As expected, patients with TBI had sustained a higher overall magnitude of injury as compared to patients without TBI (*P *< 0.001). Patients without relevant TBI and a high FFP:pRBC ratio were slightly younger and more frequently male than patients who received a low FFP:pRBC ratio transfusion. Physiological variables such as heart rate and blood pressure both at the trauma scene and upon ER arrival were comparable between all groups. Patients in the low FFP:pRBC ratio groups were slightly more shocked than patients with a high FFP:pRBC ratio upon ER admission as reflected by lower base excess. The volume loading during the prehospital and early in-hospital phases of care did not differ among the groups, except for the amount of crystalloids in the ER in patients with TBI (Table 2). Among all subgroups, the vast majority (>80%) of patients presented with manifest clinical and laboratory signs of coagulopathy upon ER arrival (Table 1). The number of transfused pRBC units was comparable across all subgroups, while the amount of plasma transfused differed considerably between the groups according to the definition given in Table 2.

**Table 1:** Basic characteristics and physiological data of patients according to their study groups^a^

	AIS score, head <3			AIS score, head ≥3		
	
Patient characteristics	FFP:pRBC ratio ≤1:2	FFP:pRBC ratio >1:2	*P *value	FFP:pRBC ratio ≤1:2	FFP:pRBC ratio >1:2	*P *value
Number of patients (%)	212 (17%)	426 (34.1%)		167 (13.4%)	445 (35.6%)	
Mean age, yr (±SD)	45.9 ± 20	42.0 ± 17.2	0.049	40.5 ± 19.2	40.2 ± 18.2	0.947
Males, *n *(%)	149 (70.3%)	330 (77.5%)	0.048	111 (66.5%)	314 (70.6%)	0.327
Blunt trauma mechanism, *n *(%)	191 (90.1%)	379 (89%)	0.722	158 (94.6%)	417 (93.7%)	0.676
Mean ISS, points (±SD)	36.7 ± 15.3	35.4 ± 13.5	0.532	49.5 ± 14.9	47.2 ± 14.1	0.143
Mean GCS at trauma scene, points	10	12	0.001	7	7	0.571
Mean HR at trauma scene, beats/min (±SD)	105 ± 31	106 ± 25	0.907	106 ± 33	105 ± 28	0.240
Mean HR at hospital admission, beats/min (±SD)	105 ± 27	102 ± 26	0.222	103 ± 31	104 ± 26	0.686
Mean systolic BP at trauma scene, mmHg (±SD)	91 ± 34	95 ± 31	0.245	90 ± 35	93 ± 35	0.792
Mean systolic BP at hospital admission, mmHg (±SD)	93 ± 36	95 ± 30	0.411	92 ± 34	96 ± 30	0.313
Mean Hb, g/dl (±SD)	8.0 ± 2.7	8.4 ± 2.8	0.09	8.0 ± 2.9	8.4 ± 3.0	0.13
Mean BE, mM/l (±SD)	-8.9 ± 6.8	-7.0 ± 5.9	0.08	-9.3 ± 6.5	-7.3 ± 6.4	0.01
Mean PTT, seconds (±SD)	51.9 (±32.8)	50.9 (±31.1)	0.63	72.3(±49.3)	60.7(±37.5)	0.06
Mean quick, % (±SD)	54 (±23.7)	56 (±23.4)	0.33	54 (±24.2)	53 (±23.0)	0.69
Mean platelets, nl (±SD)	158 (±77.7)	165 (±75.6)	0.30	152 (±74.0)	160 (±71.6)	0.23
Coagulopathy, *n *(%)	152 (87.4%)	298 (82.3%)	0.14	118 (90.1%)	344 (88.4%)	0.61

^a^AIS, Abbreviated Injury Scale; FFP, fresh frozen plasma; pRBC, packed red blood cell; SD, standard deviation; ISS, Injury Severity Score; GCS, Glasgow Coma Scale; HR, heart rate; BP, blood pressure; Hb, hemoglobin; BE, base excess; PTT, partial thromboplastin time. Coagulopathy was defined by the presence of at least one of the following conditions: PTT <38 seconds, Quick <70% and platelets <100,000/μl. In the TR-DGU the prothrombin time (PT) is documented as Quick's value where a value of <70% is equivalent to an INR (International Normalized Ratio) > 1.3.

**Table 2:** Fluids and blood products transfused during initial resuscitation^a^

	AIS score, head <3			AIS score, head ≥3		
	
Transfusions and time intervals	FFP:pRBC ratio ≤1:2	FFP:pRBC ratio >1:2	*P *value	FFP:pRBC ratio ≤1:2	FFP:pRBC ratio >1:2	*P *value
Fluids prehospital						
Mean crystalloids, ml (±SD)	1,491 ± 937	1,391 ± 819	0.433	1,354 ± 888	1,297 ± 743	0.981
Mean colloids, ml (±SD)	991 ± 615	939 ± 579	0.272	982 ± 654	945 ± 475	0.957
Fluids emergency room						
Mean crystalloids, ml (±SD)	3,549 ± 2,858	3,981 ± 2,959	0.071	3,122 ± 2,640	4,000 ± 3,036	<0.001
Mean colloids, ml (±SD)	1,648 ± 1,478	1,802 ± 1,592	0.080	1,568 ± 1,247	1,788 ± 1,371	0.112
Mean pRBC transfusion, *n *(±SD)	19.5 ± 11.2	19.5 ± 11.9	0.916	18.4 ± 9.8	18.9 ± 10.7	0.980
Mean FFP transfusion, *n *(±SD) (min-max)	5.7 ± 5.2 (0 to 32)	18.0 ± 12.3 (6 to 88)	<0.001	5.5 ± 4.8 (0 to 30)	17.8 ± 10.4 (6 to 84)	<0.001
Mean minutes from trauma scene to ER (±SD)	59 (±39)	64 (±40)	0.115	66 (±44)	69 (±44)	0.464
Mean minutes from hospital admission including ER and operation theater to ICU transfer (±SD)	303 (±144)	350 (±140)	<0.001	290 (±147)	337 (±143)	0.001

^a^AIS, Abbreviated Injury Scale; FFP, fresh frozen plasma; pRBC, packed red blood cell; SD, standard deviation; ER, emergency room; ICU, intensive care unit.

**Figure 2:**
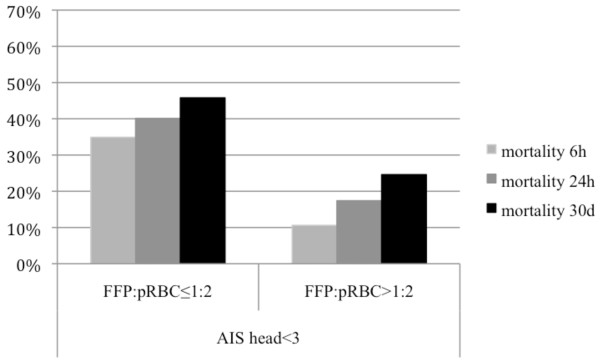
**Mortality among non-traumatic brain injury (non-TBI) patients according to fresh frozen plasma to packed red blood cell (FFP:pRBC) ratios transfused**. Mortality rates are shown at 6 hours, 24 hours and 30 days for severely injured patients without TBI (Abbreviated Injury Scale (AIS) score, head <3) who had received a massive transfusion with a high or low FFP:pRBC ratio.

**Figure 3:**
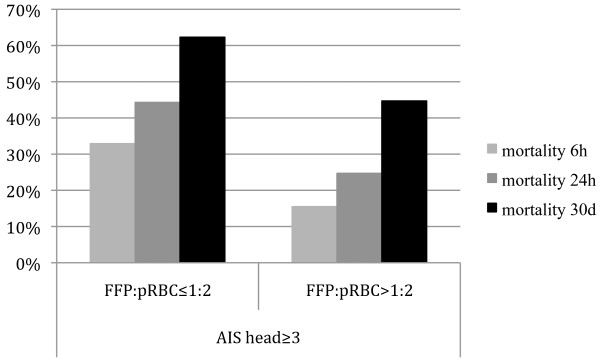
**Mortality for traumatic brain injury (TBI) patients according to fresh frozen plasma to packed red blood cell (FFP:pRBC) ratios transfused**. Mortality rates are shown at 6 hours, 24 hours and 30 days for severely injured patients with TBI (Abbreviated Injury Scale (AIS) score, head ≥3) who had received a massive transfusion with a high or low FFP:pRBC ratio.

**Figure 4:**
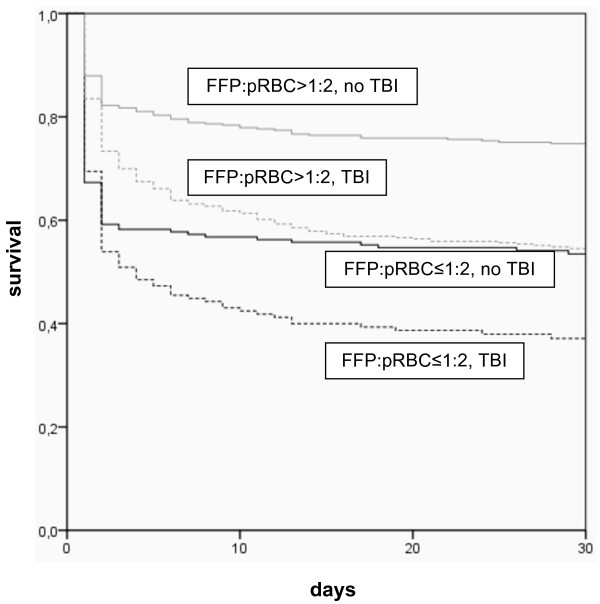
**The 30-day survival rates for patients with different transfusion regimes subdivided into patients with and without traumatic brain injury (TBI)**. Kaplan-Meier analysis was performed to assess the outcomes of severely injured patients who had received a high FFP:pRBC ratio transfusion (FFP:pRBC >1:2) versus those who had received a low FFP:pRBC ratio transfusion (FFP:EK ≤1:2), subdivided into patients with TBI (Abbreviated Injury Scale (AIS) score, head ≥3) and patients without TBI (AIS score, head <3).

In general, the mortality rate was lower in the high FFP:pRBC ratio groups as compared to the low FFP:pRBC ratio groups, regardless of the presence or absence of TBI and across all time points (Table 3 and Figures 2 and 3). Figure 4 shows Kaplan-Meier 30-day survival curves for all subgroups, with the highest survival rate being in patients without TBI who received a high FFP:pRBC ratio. The lowest survival rates were found in patients with TBI who were transfused with a low FFP:pRBC ratio. The differences in survival between the high and low FFP:pRBC ratio groups were statistically significant within each TBI subgroup (*P *< 0.001; log-rank test). The frequency of sepsis and multiple organ failure did not differ among the groups, except for sepsis in patients with TBI who were transfused with a high FFP:pRBC ratio (>1:2). Other secondary end points, such as ventilator-free days, ICU length of stay and overall in-hospital length of stay differed significantly between the ratio groups, but not when analyzed for survivors only.

**Table 3:** Morbidity and mortality according to study groups^a^

	AIS score, head <3			AIS score, head ≥3		
	
Morbidity and mortality	FFP:pRBC ratio ≤1:2	FFP:pRBC ratio >1:2	*P *value	FFP:pRBC ratio ≤1:2	FFP:pRBC ratio >1:2	*P *value
Sepsis, *n *(%)	31 (21.5%)	91 (23.6%)	0.608	19 (15.7%)	98 (24.9%)	0.035
Multiorgan failure, *n *(%)	86 (58.5%)	211 (55.7%)	0.557	80 (67.2%)	276 (71.3%)	0.393
Mean ventilator-free days (±SD)	8.7 ± 11.2	12.8 ± 11.6	<0.001	4.3 ± 8.1	6.1 ± 9.0	0.006
Survivors' mean ventilation-free days (±SD)	16.9 ± 10.2	17.4 ± 10.1	0.647	11.5 ± 9.6	11.1 ± 9.7	0.825
Mean ICU LOS, days (±SD)	14.7 ± 19.4	18.5 ± 20.1	<0.001	12.5 ± 18.5	18.2 ± 21.3	<0.001
Survivors' mean ICU LOS, days (±SD)	24.7 ± 20.5	23.1 ± 20.6	0.335	29.0 ± 20.8	29.7 ± 22.3	0.703
Mean HLOS, days (±SD)	30.2 ± 40.3	43.3 ± 40.2	<0.001	20.6 ± 30.1	29.9 ± 36.4	<0.001
Survivors' mean HLOS, days (±SD)	54.3 ± 43.0	56.3 ± 38.5	0.361	49.2 ± 32.3	49.9 ± 36.8	0.934
6-hour mortality, *n *(%)	74 (34.9%)	45 (10.6%)	<0.001	55 (32.9%)	69 (15.5%)	<0.001
24-hour mortality, *n *(%)	85 (40.1%)	47 (17.4%)	<0.001	74 (44.3%)	110 (24.7%)	<0.001
30-day mortality, *n *(%)	97 (45.8%)	105 (24.6%)	<0.001	104 (62.3%)	199 (44.7%)	<0.001
In-hospital overall mortality, *n *(%)	102 (48.1%)	114 (26.8%)	<0.001	104 (62.3%)	203 (45.6%)	<0.001

^a^AIS, Abbreviated Injury Scale; FFP, fresh frozen plasma; pRBC, packed red blood cell; ICU, intensive care unit; LOS, length of stay; SD, standard deviation; HLOS, hospital length of stay.

Multivariate logistic regression analysis was performed according to the presence or absence of TBI with hospital mortality as the dependent variable. The analysis identified a high FFP:pRBC transfusion ratio (>1:2) as an independent predictor for survival in both subgroups (Table 4). Interestingly, severely injured patients who had sustained a TBI obviously benefited more from the high FFP:pRBC ratio strategy as compared to patients without TBI (ORs, 0.48 vs. 0.70). In both groups, the RISC score was independently associated with mortality. Multivariate analysis was also used to calculate all RISC score variables entered into the model, instead of the cumulative score, which yielded the same results. The additional consideration of emergency craniotomy in the TBI group did not improve the model (OR, 1.1; *P *= 0.6).

**Table 4:** Logistic regression analysis^a^

Non-TBI versus TBI patients	Regression coefficient β	Standard error of the mean	*P *value	Odds ratio (95% CI)
No TBI				
FFP:pRBC ratio >1:2	-0.36	0.28	0.200	0.70 (0.04 to 1.21)
RISC score	0.75	0.09	<0.001	2.13 (1.77 to 2.56)
Emergency operation	0.09	0.30	0.759	1.10 (0.61 to 1.98)
Constant	0.02	0.26	0.947	1.02
TBI				
FFP:pRBC ratio >1:2	-0.73	0.27	0.006	0.48 (0.29 to 0.81)
RISC score	0.56	0.07	<0.001	1.75 (1.52 to 2.02)
Emergency operation	0.45	0.29	0.112	1.57 (0.90 to 2.75)
Constant	0.14	0.24	0.543	1.16

^a^TBI, traumatic brain injury; 95% CI, 95% confidence interval; FFP, fresh frozen plasma; pRBC, packed red blood cell; RISC, Revised Injury Severity Classification. Logistic regression analysis was performed with mortality as a dependent variable and FFP:pRBC ratio, RISC score and emergency operations as independent variables according to the presence or absence of TBI.

## Discussion

Although there is still debate about the early and aggressive use of FFP in a more balanced 1:1 ratio to pRBCs to correct ATC, the majority of studies published to date have emphasized its use and have associated this approach with improved survival rates among severe trauma patients requiring massive transfusions [4,6-11,15,16]. These studies, however, have not attempted to determine whether TBI patients have the same association of improved survival with a more balanced transfusion of FFP and pRBCs. In patients with TBI, there is a component of local tissue release of thromboplastin and low platelets. In the present study, we retrospectively investigated whether a high FFP:pRBC transfusion ratio (FFP:pRBC ratio >1:2) during massive transfusion (≥10 U of pRBCs) in the acute phase after trauma would be associated with a similar survival benefit in patients with TBI as was previously observed for patients without TBI.

As we describe in the Results, the rates for acute and late mortality were both lower in high FFP:pRBC ratio groups compared to low FFP:pRBC ratio groups, regardless of the presence or absence of TBI. Despite the fact that mortality rates in patients with TBI were generally higher compared to patients without TBI, obviously due to the higher magnitude of overall injury sustained, this result indicates that the concept of a high transfusion ratio approach in the acute trauma setting requiring massive transfusion may also be beneficial for patients with accompanying TBI. This assumption is further supported by the results from our logistic regression analysis, which identified a high FFP:pRBC ratio to be an independent predictor for survival, especially in TBI patients. As the high FFP:pRBC ratio effect on survival has been observed as an early effect, immediate introduction of this approach after ER admission may be of crucial importance as soon as clinical and laboratory signs indicate an individual at risk for massive transfusion. To support the clinician, several scoring systems to enable early identification of patients at risk for massive transfusion and to predict the probability for massive transfusion at a very early stage have been developed and validated, such as the Assessment of Blood Consumption score or the predictive model for massive transfusion described by Cotton *et al*. [17] and McLaughlin *et al*. [18]. Recently, our group [19,20] has introduced the Trauma Associated Severe Hemorrhage score as a useful instrument to calculate the probability of massive transfusion as a surrogate for life-threatening hemorrhage after severe trauma.

Patients with TBI are at increased risk for developing coagulation abnormalities in the acute phase after trauma, and the clinical frequency of these disorders in blunt TBI patients has been estimated to be as high as 22.7% [12]. The coagulopathy after TBI is thought to be the result of injury-mediated local release of thromboplastin into the systemic circulation, although the precise mechanisms that cause systemic coagulopathy after TBI are as yet unknown [12,21,22]. In 1979, Mauersberger [23] concluded on the basis of his clinical investigations that the release of tissue thromboplastin after severe brain injury can lead to a consumption coagulopathy. Hulka *et al*. [24] reported a similar coagulopathy after blunt TBI that was associated with a high frequency of death. These observations regarding the early substitution of plasma have been advocated. More recently, Cohen *et al*. [25] emphasized the role of hypoperfusion and the protein C pathway for the development of coagulopathy after TBI. These authors concluded that TBI alone does not initiate coagulopathy, but must be coupled with hypoperfusion to lead to coagulation derangement associated with the activation of the protein C pathway.

One criticism associated with transfusion of high volumes of FFP during early acute resuscitation after trauma has been related to its potential harmful effects [26]. Potential risks of FFP transfusion include transfusion-related acute lung injury (TRALI) [27], transfusion-associated circulatory overload [28], allergic reaction and transmission of infectious diseases such as human immunodeficiency virus, hepatitis B and C viruses and prion diseases [26]. To date, TRALI is the most important cause of transfusion-associated morbidity and mortality [29]. In previous retrospective studies, a trend was observed in that the survival benefit associated with the high FFP:pRBC ratio approach was bought on the account of increasing frequencies of complications, such as nosocomial infections, sepsis and organ failure, resulting in prolonged days on ventilators and increased ICU and overall in-hospital stays [16,30]. When considering all patients included in the present study, survivors' and nonsurvivors' duration of ventilation, ICU length of stay and HLOS were significantly longer for patients who had received a high FFP:pRBC transfusion ratio. This observation might be due to prolonged survival among patients with a high FFP:pRBC transfusion ratio. When focusing on survivors only, the duration of ventilation, ICU length of stay and HLOS did not differ statistically between the high and low FFP:pRBC transfusion ratio groups. The incidence of multiple organ failure was higher in patients with more severe injuries, including TBI, than in patients without TBI (67% and 71% versus 59% and 56% according to the transfusion ratios, respectively), but did not differ when the transfusion regimens within the two subgroups were compared. Patients with TBI who received a high FFP:pRBC transfusion ratio developed septic coagulation more often than patients of the same group but with a low FFP:pRBC transfusion ratio.

The present observations are limited by the number of included patients and the study's retrospective design. It should also be mentioned that the observed positive effect of the high FFP:pRBC transfusion ratio could be explained at least in part by a selection bias. This means that patients in whom it was possible to administer large amounts of plasma in parallel to pRBC transfusion did not show a dramatic massive blood loss, which would require quick transfusion of large amounts of blood. Administration of FFP usually requires some time for preparation. To minimize this survivorship bias, we excluded *a priori *patients who died within the first hour after admission and limited the duration of transfusion to initial resuscitation; that is, only blood products were considered that had been transfused between the time of ER arrival and ICU admission. Snyder *et al*. [31] additionally included the FFP:pRBC transfusion ratio as a time-dependent covariate in their multivariate analysis. However, since the exact timing of transfusion is not documented in the TR-DGU, and only the cumulative amount of transfused blood products between the time of ER arrival and ICU admission is recorded, we were not able to adjust for survivorship bias in our logistic regression model as was done by Snyder *et al*. [31]. Furthermore, this study is based on registry data only and is thus limited by the lack of a more detailed description of the coagulation management that was performed, for example, platelet transfusion or the administration of other specific coagulation factors. In this context, Perkins *et al*. [32,33] and Holcomb *et al*. [4] previously described the association between the amount of transfused platelets and improved survival. Future research should be conducted with larger patient numbers and using a prospective approach to prove these results.

## Conclusions

Despite the ongoing debate with regard to the early, aggressive use of FFP in a more balanced 1:1 ratio to pRBCs in the acute trauma setting to correct coagulation disorders, the present study provides some more detailed information in favor of this approach. The mortality rates were consistently lower in the high FFP:pRBC transfusion ratio groups versus the low FFP:pRBC transfusion ratio groups, regardless of the presence or absence of TBI and at all time points studied, indicating that the concept of a high FFP:pRBC transfusion ratio may also be valid for patients with TBI. Regarding survivors, morbidity was comparable for patients with a low or high FFP:pRBC transfusion ratio, regardless of the presence or absence of TBI.

## Key messages

• Retrospective studies have demonstrated a potential survival benefit from transfusion strategies using an early and more balanced ratio between FFP and pRBC transfusions in patients with ATC requiring massive transfusion but results have mostly been derived from non-head injured patients.

• A total of 1,250 data sets of severely injured patients from the TR-DGU from 2002 to 2008 were analyzed to determine whether a transfusion regime using a high FFP:pRBC ratio (FFP:pRBC >1:2) would be associated with a similar survival benefit in severely injured patients with TBI (AIS score, head ≥3) as demonstrated for patients without TBI requiring massive transfusion (≥10 U of pRBCs).

• The mortality rates were consistently lower in the high-ratio groups versus the low-ratio groups, regardless of the presence or absence of TBI and at all time points studied (*P *< 0.001).

• The concept of a high FFP:pRBC ratio may also be valid for patients with TBI. Multivariate logistic regression analysis identified a high FFP:pRBC ratio as an independent predictor of survival, especially in TBI patients.

• Future research should be conducted with a larger number of patients to prove these results in a prospective study.

## Abbreviations

AIS: Abbreviated Injury Scale; ATC: acute traumatic coagulopathy; CI: confidence interval; ER: emergency room; FFP: fresh frozen plasma; HLOS: in-hospital length of stay; ICU: intensive care unit; ISS: Injury Severity Scale; LOS: length of stay; OR: odds ratio; pRBC: packed red blood cell; TACO: transfusion-associated circulatory overload; TBI: traumatic brain injury; TRALI: transfusion-related acute lung injury; TR-DGU: TraumaRegistry of the *Deutsche Gesellschaft für Unfallchirurgie*.

## Competing interests

The authors declare that they have no competing interests.

## Authors' contributions

SP and MM conceived the study. UN and RL undertook the statistical analysis together with MB and AW. All other authors (SW, MB and PS) contributed to the study design and to data sharing. SP and MM were responsible for writing the manuscript. All authors read and approved the final manuscript.
